# Structural modeling of an outer membrane electron conduit from a metal-reducing bacterium suggests electron transfer via periplasmic redox partners

**DOI:** 10.1074/jbc.RA118.001850

**Published:** 2018-04-10

**Authors:** Marcus J. Edwards, Gaye F. White, Colin W. Lockwood, Matthew C. Lawes, Anne Martel, Gemma Harris, David J. Scott, David J. Richardson, Julea N. Butt, Thomas A. Clarke

**Affiliations:** From the ‡ Centre for Molecular and Structural Biochemistry, School of Biological Sciences and School of Chemistry, University of East Anglia, Norwich NR4 7TJ, United Kingdom,; § Institut Laue-Langevin, 38042 Grenoble, France,; ¶ Research Complex at Harwell, Rutherford Appleton Laboratory, Oxfordshire OX11 0FA, United Kingdom,; ‖ ISIS Spallation Neutron and Muon Source, Rutherford Appleton Laboratory, Oxfordshire OX11 0QX, United Kingdom,; ** School of Biosciences, University of Nottingham, Sutton Bonington Campus, Leicestershire LE12 5RD, United Kingdom

**Keywords:** cytochrome, membrane protein, protein complex, electron transfer complex, liposome, shewanella, outer membrane, small-angle neutron scattering, MtrCAB

## Abstract

Many subsurface microorganisms couple their metabolism to the reduction or oxidation of extracellular substrates. For example, anaerobic mineral-respiring bacteria can use external metal oxides as terminal electron acceptors during respiration. Porin–cytochrome complexes facilitate the movement of electrons generated through intracellular catabolic processes across the bacterial outer membrane to these terminal electron acceptors. In the mineral-reducing model bacterium *Shewanella oneidensis* MR-1, this complex is composed of two decaheme cytochromes (MtrA and MtrC) and an outer-membrane β-barrel (MtrB). However, the structures and mechanisms by which porin–cytochrome complexes transfer electrons are unknown. Here, we used small-angle neutron scattering (SANS) to study the molecular structure of the transmembrane complexes MtrAB and MtrCAB. *Ab initio* modeling of the scattering data yielded a molecular envelope with dimensions of ∼105 × 60 × 35 Å for MtrAB and ∼170 × 60 × 45 Å for MtrCAB. The shapes of these molecular envelopes suggested that MtrC interacts with the surface of MtrAB, extending ∼70 Å from the membrane surface and allowing the terminal hemes to interact with both MtrAB and an extracellular acceptor. The data also reveal that MtrA fully extends through the length of MtrB, with ∼30 Å being exposed into the periplasm. Proteoliposome models containing membrane-associated MtrCAB and internalized small tetraheme cytochrome (STC) indicate that MtrCAB could reduce Fe(III) citrate with STC as an electron donor, disclosing a direct interaction between MtrCAB and STC. Taken together, both structural and proteoliposome experiments support porin–cytochrome–mediated electron transfer via periplasmic cytochromes such as STC.

## Introduction

A broad range of subsurface microorganisms couple their metabolism to the reduction or oxidation of extracellular substrates. Dissimilatory metal-reducing bacteria can, in the absence of oxygen, utilize metal oxides as terminal electron acceptors during respiration. Iron-oxidizing bacteria generate energy by coupling the oxidation of Fe(II) to the reduction of oxygen, enabling aerobic bacteria to survive in nutrient-limited environments. For Gram-negative bacteria, these processes require electrons to be channeled across the outer membrane, which is achieved through the assembly of porin–cytochrome complexes that form within the outer membrane (1, 2).

The number of identified porin–cytochrome complexes is rapidly growing. Gene clusters with adjacent genes encoding a β-barrel porin and multiheme cytochromes have been identified in a broad range of bacteria, including the lithoautotroph *Sideroxydans lithotrophicus* ES-1, phototrophic *Rhodopseudomonas palustris* TIE-1 (3, 4), and complete complexes from *Geobacter sulfurreducens* have been isolated (5). However, the best-studied of these complexes is the MtrCAB porin–cytochrome complex from the facultative anaerobe *Shewanella oneidensis* MR-1, a model organism for the study of the reduction of extracellular minerals and metals (6). The MtrCAB complex consists of a periplasmic decaheme cytochrome MtrA, a transmembrane 28-strand β-barrel MtrB, and a second decaheme cytochrome MtrC on the cell surface. It is proposed that MtrA enters the periplasmic side of MtrB, and MtrC inserts into the extracellular side of MtrB (Fig. S1*A*). The positions of the two cytochromes inside MtrB are close enough to enable electron exchange, allowing electrons to move through a chain of hemes from the periplasm to the surface (7). The outer membrane cytochromes OmcA and MtrC are then responsible for mediating electron transfer from the cell surface to terminal electron acceptors (8–10, 12).

A structural model of MtrA was experimentally analyzed using small-angle X-ray scattering (SAXS), which resulted in a molecular envelope of MtrA resembling a paddle-like shape with approximate dimensions of 100 × 25 × 50 Å; it was suggested that the narrow end of MtrA could insert into MtrB, with most of MtrA accessible for electron exchange with periplasmic electron acceptors (13). MtrB is predicted to be a 28-strand outer membrane β-barrel protein with an N-terminal domain of only ∼30 amino acids. Unlike typical outer membrane β-barrels, MtrB is oriented in the membrane so that the soluble loops that link each β-strand are shorter on the cell surface, whereas the longer solvent-exposed loops remain in the periplasm (12). This suggests that MtrB has a different folding pathway to other transmembrane β-barrel proteins, and may be linked to the interaction between MtrA and MtrB during folding.

The crystal structure of a soluble form of MtrC is available (14). MtrC is composed of four domains, with the hemes bound in two pentaheme domains and the other two domains forming β-barrels that support the pentaheme domains (Fig. S1*B*). The hemes are arranged in a staggered cross conformation, giving four possible sites for electrons to enter or exit MtrC. Two sites are at the edges of the pentaheme domains, whereas two others face into the β-barrel domains. It is not yet clear which of these MtrC sites would accept electrons from MtrA and which sites transfer electrons into terminal extracellular acceptors.

Electron transfer to MtrCAB depends on CymA, a tetraheme menaquinol dehydrogenase in the cytoplasmic membrane, which can reduce a range of soluble periplasmic cytochromes (15). These include the small tetraheme cytochrome (STC)^3^ and tetraheme fumarate reductase FccA, as well as monoheme ScyA and the diheme ccp peroxidase (16). Both purified STC and FccA are capable of transferring electrons when mixed with purified MtrA, but this has never been demonstrated with membrane-bound MtrCAB complexes. It has been proposed that MtrA can directly exchange electrons with CymA, allowing static or dynamic electron transfer networks to assemble between CymA and the porin–cytochrome complex MtrCAB in the outer membrane of the cell (16–18).

There is no experimental evidence for the overall shape of the MtrCAB complex, or exactly how MtrA and MtrC interact with MtrB. These data are important to understanding the mechanism of MtrCAB, including 1) how far MtrA can extend into the periplasm and interact with electron donors, 2) how far both MtrA and MtrC extend into MtrB, and 3) the orientation of MtrC on the surface of the complex and the cell. We sought to use small-angle scattering methods to obtain low-resolution information about the structural configuration of MtrAB and MtrCAB and address many of the structural queries that challenge our understanding of this complex. In this project, we used small-angle neutron scattering (SANS) to derive *ab initio* molecular envelope models of MtrCAB and MtrAB. SANS is a useful technique to help elucidate the overall domain organization of membrane proteins, as proteins and detergent molecules have different neutron-scattering lengths. This enables “contrast matching,” where the scattering length of the solvent is adjusted to match the scattering length of the detergent; this is enabled by altering the ratio of D_2_O/H_2_O in the solvent. At this contrast match point the scattering contribution of detergent is the same as the solvent and so only the scattering of the protein is observed. This allows structural information about the protein derived independent of contributions from the supporting detergent micelle. Using this technique, *ab initio* molecular models were derived that provided valuable structural information about the organization of the MtrCAB component proteins, as well as the likely electron ingress and egress routes out of the complex and the overall structural arrangement in the membrane. These results led us to conclude that MtrCAB must interact with soluble periplasmic cytochrome electron shuttles rather than directly with the inner membrane CymA and to test this we constructed an artificial model of the *Shewanella* periplasmic electron transfer system using MtrCAB proteoliposomes with internalized STC and external iron citrate.

## Results

### SANS and modeling of the porin–cytochrome complexes MtrAB and MtrCAB

Native MtrCAB and recombinant MtrAB were isolated from *S. oneidensis* as described in supporting data and SDS-PAGE was used to confirm purity and presence of component proteins in both MtrAB and MtrCAB (Fig. S2). Both proteins were buffer exchanged into 20 mm HEPES, pH 7.8, 100 mm NaCl, 2.8 mm Fos–choline-12, 13% D_2_O directly before SANS measurements.

**Figure 1.**
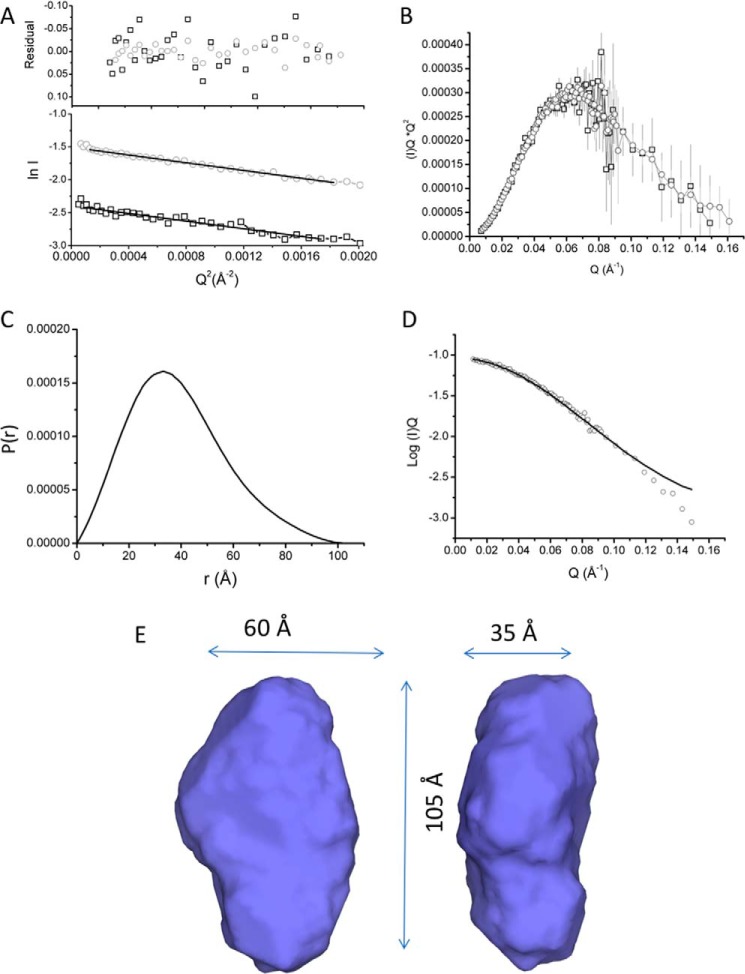
**Small angle neutron scattering of MtrAB in 20 mm HEPES pH 7.8, 100 mm NaCl, 13% D_2_O, 2.8 mm Fos-choline-12.** *A*, Guinier region of MtrAB at 3.5 (*black*) and 8.7 (*gray*) mg/ml. Lines are linear fits used to calculate *R_g_.* Residuals of fit are shown above experimental curve. *B*, Kratky plot of scaled and overlaid 3.5 (*black*) and 8.7 (*gray*) mg/ml. *C*, *P*(*r*) distance distribution curve of merged MtrAB data sets. *D*, scattering curve of merged 3.5 and 8.7 mg/ml datasets. The line is a fit of the *P*(*r*) curve shown in (*C*) to the scattering data. *E*, molecular envelope generated by DAMFILT, representing the core features of 19 independent models selected from 20 generated models fitted to the experimental data.

Initial SANS characterization was carried out on SANS2D (ISIS, Oxfordshire, UK) where the monodispersity of samples were confirmed via Guinier analysis over a range of concentrations. Further data for shape reconstruction was subsequently obtained on D22 (Institut Laue-Langevin (ILL), Grenoble, France) using MtrAB diluted to 3.5 and 8.7 mg/ml of protein in 20 mm HEPES, pH 7.8, 100 mm NaCl, 2.8 mm Fos–choline-12, 13% D_2_O. Guinier analysis of MtrAB at both concentrations indicated that the samples were not aggregated and had a similar radius of gyration (*R_g_*) of 29 Å (Fig. 1*A*). A Kratky plot (*I*_(_*_Q_*_)_*Q^2^ against *Q*, where *Q* is momentum transfer (19)) showed MtrAB was globular and the scattering curves of both concentrations were superposable (Fig. 1*B*), validating the absence of interparticle interactions. The two scattering curves were merged for further analysis, providing high reliability at low angle and a high signal-to-noise ratio at high *Q* values. The GNOM program (20) was used to generate *P*(*r*) distance distribution curves based on inverse Fourier transform of the data to a maximum *Q* value of 0.15 Å^−1^. Beyond this range, the scattering intensity decreased significantly so data were truncated to this value before *P*(*r*) distance distribution curves were calculated. The *P*(*r*) curve shape generated from the scattering curve was suggestive of a globular protein with an *R_g_* of 29.3 ± 0.3 Å (Fig. 1, *C* and *D*), in good agreement with the value determined by Guinier analysis. The molecular mass was calculated using the estimated *I*(0) for each concentration, giving values of 104 kDa and 103 kDa for 3.5 and 8.7 mg/ml concentrations, respectively, close to the calculated heterodimeric molecular mass of 110 kDa for MtrAB (Table S1) (21).

Pair distance distribution function (*P*(*r*)) curves were generated with the maximum distance in the molecule, *D*_max_, values ranging between 98 and 110 Å and used to generate 20 *ab initio* models using DAMMIF for each *P*(*r*) curve. The resulting models were filtered to a core of 17–19 models using DAMFILT, and averaged using DAMAVER (Table S2). The resulting model was refined directly against scattering data using a final round of DAMMIN to generate a final model. Based on the error estimates obtained at each *D*_max_ value, the best model was found at a *D*_max_ of 104 Å from the probability distribution (Table S2). The averaged model had a normalized spatial discrepancy of 0.530 ± 0.02 and the final DAMMIN model had a χ^2^ value of 0.548 (Fig. S3). The filtered model shown in Fig. 1*E* contains the core structural model conserved in all averaged models, and had approximate overall dimensions of 105 × 60 × 35 Å. The envelope is consistent with a monomeric single domain protein, although the model is ellipsoidal, rather than cylindrical, suggesting that the barrel is not completely symmetrical. The experimental length of MtrAB obtained in these studies is approximately the same as the length of MtrA (13), and so these results are consistent with an MtrAB model where MtrA is fully inserted into the MtrB β-barrel.

**Figure 2.**
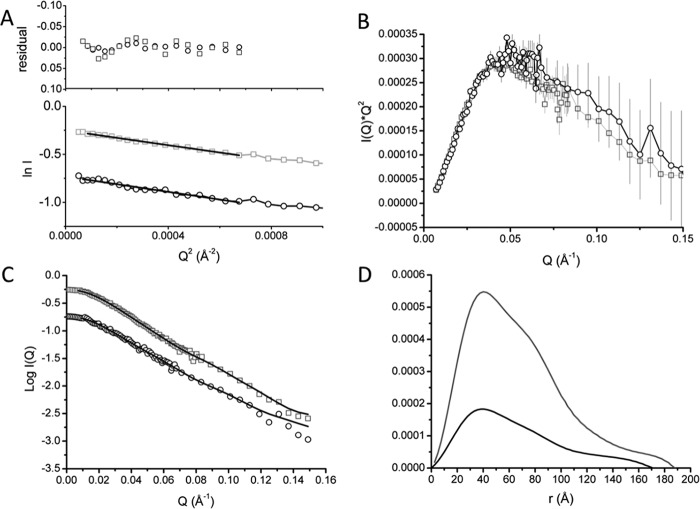
**SANS data analysis of MtrCAB in 20 mm HEPES pH 7. 8, 100 mm NaCl, 13% D_2_O, 2.8 mm Fos-choline-12, at 3.5 (*black*) and 9.7 (*gray*) mg/ml.** *A*, Guinier region of MtrCAB with linear fit to data shown suggesting an *R_g_* of 54 Å. Residuals of fit are shown above. *B*, Kratky plot of scaled MtrCAB suggests slight differences between the data at 3.5 and 9.7 mg/ml. *C* and *D*, scattering curves with fits to the data giving rise to the *P*(*r*) distance distribution curves shown in (*D*).

MtrCAB was diluted to 3.5 and 9.7 mg/ml in 20 mm HEPES, pH 7.8, 100 mm NaCl, 13% D_2_O, 2.8 mm Fos–choline-12 and SANS data were collected on D22 (ILL). Guinier analysis of the MtrCAB scattering curves at both concentrations gave similar *R_g_* of 53 Å, suggesting that both 3.5 and 9.7 mg/ml samples were not aggregated over this concentration range (Fig. 2*A*).

The molecular size of MtrCAB was calculated using the *I*(0) determined at each concentration (21). Molecular masses of 183 kDa and 203 kDa were calculated at 3.5 and 9.7 mg/ml MtrCAB concentration, respectively, consistent with a 1:1:1 MtrCAB complex. To confirm the molecular mass of MtrCAB solubilized in Fos–choline-12, sedimentation velocity experiments were performed on samples of MtrCAB in 2.8 mm Fos–choline-12, 100 mm NaCl, 51% D_2_O (Fig. S4). At this D_2_O concentration, the buffer density is approximately equal to the reciprocal of the Fos–choline-12 partial specific volume (22). The major sedimenting component observed has a molecular mass of 200 kDa, similar to the molecular mass of 189 kDa predicted for MtrCAB. This agrees with previous studies, which determined a 1:1:1 stoichiometry for MtrCAB (7, 23). A second minor peak had a molecular mass of 70 kDa, close to the molecular mass of MtrC and suggesting some degradation of the complex, most likely because of the instability of MtrCAB in 51% D_2_O.

The Kratky plot of MtrCAB SANS data showed a broader peak than MtrAB (Fig. 2*B*), suggesting an elongated multidomain complex. The scattering intensity decreased significantly beyond a *Q* value of 0.15, so data were truncated to this value before *P*(*r*) distance distribution curves were calculated. Despite attempts to adjust the data using buffer subtraction, there was a slight difference in *I*(*Q*)*Q^2^ observed after 0.05 Å^−1^. This may be caused by interparticle interactions at higher concentrations, or by weaker scattering at lower concentrations. The scattering curves of the two concentrations were therefore processed separately.

*P*(*r*) distance distribution plots produced *R_g_* values of 53.7 ± 0.2 Å and 52.7 ± 0.6 Å at 3.5 and 9.7 mg/ml, respectively, and initial *D*_max_ values of 174 and 185 Å. The increase in *P*(*r*) at the higher distance range shape of the curves for both concentrations suggests that the association of MtrC to MtrAB results in a different, more substantially elongated complex, which is consistent with the Kratky plots (Fig. 2, *C* and *D*). Both concentrations were analyzed across *D*_max_ values of 150 and 190 Å to determine the most probable model for the two concentrations (Table S2). Quality estimates from data processed using GNOM suggested that the optimal *D*_max_ was between 165 and 180 Å, and the χ^2^ values for final DAMMIN models were also highest at this range. Irrespective of the initial *D*_max_ predicted from *P*(*r*) plots, the maximum edge-to-edge distance between atoms in the final DAMMIN model was slightly higher, with values of 180–185 Å obtained for both concentrations.

**Figure 3.**
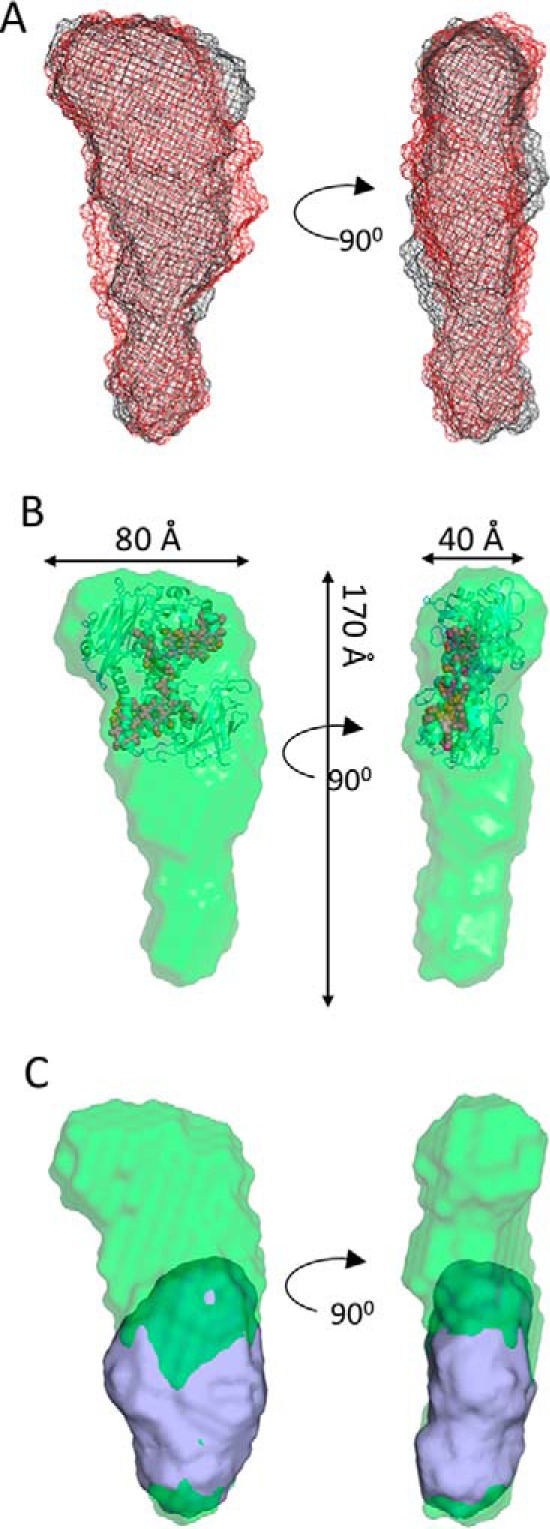
**MtrCAB molecular envelopes generated from DAMFILT from multiple models.** *A*, DAMFILT molecular envelopes generated from scattering data of MtrCAB at 3.5 mg/ml (*black mesh*) and 9.7 mg/ml (*red mesh*). *B*, DAMFILT molecular envelope of MtrCAB generated by averaging all models generated at 3.5 and 9.7 mg/ml. The crystal structure of MtrC (PDB ID: 4LM8) is superposed using SUPCOMB into the MtrCAB envelope. The heme groups are shown as *red spheres. C*, superposition of MtrCAB and MtrAB molecular envelopes generated by DAMFILT and SUPCOMB.

The molecular envelopes shown in Fig. 3*A* represent the superposed filtered models of MtrCAB at both 9.7 and 3.5 mg/ml. Despite the differences revealed in both Kratky and *P*(*r*) distance distribution plots, the two structural models are broadly similar and can be defined into two regions, an oblate “head” domain and a prolate “tail” domain. An averaged model from both concentrations was generated by filtering all 40 models generated for both concentrations using DAMFILT. A total of 38 models with a normalized spatial discrepancy of 0.713 ± 0.06 were averaged using DAMAVER, and the filtered core, with a length of 170 Å, is shown in Fig. 3*B*. The overall dimensions of the head domain are ∼80 × 50 × 40 Å and the prolate tail domain has approximate dimensions of 110 × 60 × 40 Å. The dimensions of the head domain are in broad agreement with the known dimensions of the MtrC crystal structure (90 × 60 × 40 Å), and the dimensions of the tail domain are consistent with the dimensions of the MtrAB SANS molecular envelope shown in Fig. 1*E*. It was possible to dock the MtrC structure into the head group of the MtrCAB filtered model using SUPCOMB (24) so that heme 10 or heme 5 is angled toward the base (Fig. 3*B*), and dock the MtrAB structural model into the remaining area of MtrCAB (Fig. 3*C*).

The level of resolution obtained for both complexes made it difficult to conclude whether the different shapes of the molecular envelopes corresponding to MtrAB and the MtrAB component complexed to MtrC were because of structural changes. However, it is possible to conclude that MtrC does not embed within MtrAB, but is docked to the surface with either heme 10 or heme 5 of MtrC within 15 Å of the surface of MtrAB, and the other heme extended away from the cell surface and into the extracellular environment toward potential terminal electron acceptors.

### Electron transfer between MtrCAB and STC

MtrCAB can be incorporated into proteoliposomes containing internalized electron mediators, allowing the study of MtrCAB-mediated transmembrane electron transport (25). To show that MtrCAB could interact with internalized soluble redox partners, we used proteoliposomes to mimic the outer membrane and to determine whether the periplasmic STC could transfer electrons to MtrCAB. The width of the lipid bilayer is ∼50 Å, so the maximum distance that the 105 Å long MtrAB could extend into the periplasm would be ∼55 Å.

**Figure 4.**
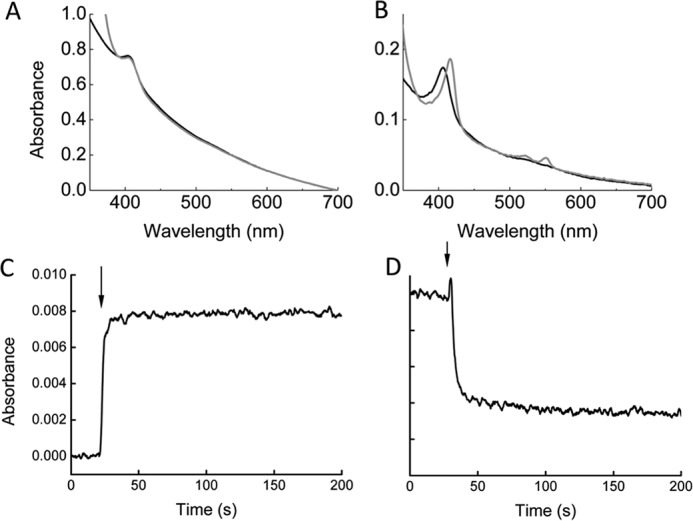
**Proteoliposome experiments using internalized STC.** *A* and *B*, UV-visible spectra of STC liposomes in presence and absence of reductant. Liposomes were suspended in 50 mm HEPES, pH 7.0, 2 mm CaCl_2_, 10 mm KCl before (*black*) and after (*gray*) incubation with 0.25 mm sodium dithionite. *A*, liposomes prepared in 0.2 mm STC without MtrCAB. *B*, proteoliposomes prepared in 0.2 mm STC and MtrCAB. *C* and *D*, time-dependent oxidation and reduction of proteoliposomes containing MtrCAB monitored by changes in UV-visible absorbance. *Arrows* show addition of reductant or oxidant at time indicated. *C*, oxidized STC proteoliposomes monitored at 550 nm on addition of excess sodium dithionite. *D*, reduced STC proteoliposomes monitored at 550 nm on addition of 300 μm Fe(III) citrate.

Liposomes containing internalized STC with or without MtrCAB were prepared as described in the supporting data, and the UV-visible absorbance spectrum of the liposome suspension was measured before and after addition of sodium dithionite as a reducing agent. The absorbance spectrum of liposomes containing STC revealed a peak at 410 nm corresponding to the Soret peak of the oxidized *c*-type heme (Fig. 4, *A* and *B*). The concentration of MtrCAB present in liposome suspensions is less than 10 nm and consequently below spectroscopically detectable limits (19, 30). Addition of sodium dithionite to liposomes without MtrCAB did not cause any observable change in the heme spectrum because the internalized cytochromes were not able to access the reductant (Fig. 4*A*). However, when sodium dithionite was added to proteoliposomes containing MtrCAB the Soret band shifted to 420 nm and the reduced αβ peaks at 520 nm and 550 nm increased, indicating the full reduction of *c*-type hemes (Fig. 4*B*). We measured the average hydrodynamic radius of these liposomes by dynamic light scattering (25) and found the radius to be 148 ± 63 nm in the absence of MtrCAB, and 152 ± 70 nm in the presence of MtrCAB. This indicates that there was no significant change in the sizes of liposomes because of the presence of MtrCAB. These results demonstrated that functional MtrCAB proteoliposomes could be prepared containing internalized soluble STC and used as model of the outer membrane electron-transfer system. The internalized cytochromes are unable to pass through the liposome membrane and cannot be reduced by external reducing agents such as sodium dithionite, unless MtrCAB is present to act as an electron-transfer protein across the liposome membrane (Fig. S5).

By monitoring the change in absorbance of proteoliposomes at 550 nm, the time-dependent oxidation and reduction of the internalized STC could be measured. Addition of sodium dithionite to proteoliposomes containing STC and MtrCAB caused a monophasic increase in absorbance at 550 nm through the reduction of STC hemes (Fig. 4*C*). Subsequent addition of membrane-impermeable Fe(III) citrate resulted in a decrease in absorbance because of oxidation of the internalized STC hemes, and confirming bidirectional electron transfer through MtrCAB (Fig. 4*D*). The rate of electron transfer from sodium dithionite to STC was 140 nm electrons s^−1^, slightly faster than the rate of electron transfer from STC to Fe(III)citrate, which was 61 nm electrons s^−1^.

These results show that when the MtrCAB complex was incorporated in a lipid bilayer, it could mediate transmembrane electron transfer between STC and external electron acceptors. The physiologically relevant STC proteoliposomes were reversibly and fully reduced and oxidized by extracellular electron donors and acceptors (Fig. S5). This electron-transfer chain indicates that, *in vivo*, reduced STC could transfer electrons to a terminal acceptor such as Fe(III) citrate through MtrCAB across the *S. oneidensis* MR-1 outer membrane.

## Discussion

Despite recent advances in characterization of the porin–cytochrome complex MtrCAB, many critical details of the conformation of the complex remained unknown. The experiments reported here reveal important new structural details of MtrCAB, including information about interactions between MtrA, MtrB, and MtrC. The length of MtrAB was ∼100 Å, the same length as isolated MtrA (13). There was little evidence of flexibility within the *P*(*r*) or Kratky plots, consistent with biochemical data that suggest MtrA and MtrB form a tight, nondissociating complex (7, 26), and *in vivo* data that show MtrA is essential for MtrB stability (7, 26). These SANS results indicate that MtrA most likely inserts through the entire length of MtrB allowing for electron transfer solely through MtrAB, although it is possible that association with MtrB alters the dimensions of MtrA measured free in solution using small-angle X-ray scattering. It is also possible that the isolated and detergent solubilized MtrCAB form determined here may not be identical to the structure of MtrCAB present in the outer membrane of *S. oneidensis.* However, there is *in vitro* and *in vivo* evidence suggesting that MtrAB is capable of electron transfer to extracellular Fe(III) citrate and this too would be consistent with an MtrAB model where MtrA spans the entire membrane (12, 27). The cross-sectional dimensions of MtrAB shown here are ∼ 35 × 60 Å, and the corresponding dimensions of MtrA are 25 and 50 Å (13), indicating that the MtrB barrel could form an ∼10 Å shell around MtrA in the membrane.

**Figure 5.**
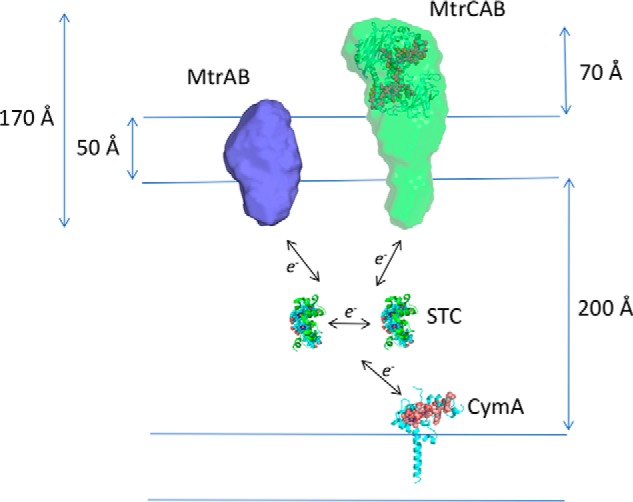
**Scale diagram of the CymA/MtrCAB electron transport system of *S. oneidensis* MR-1.** The averaged molecular envelope of MtrCAB containing the docked MtrC structure (PDB ID: 4LM8) is shown in *green*. The filtered molecular envelope model of MtrAB is shown in *blue*. The crystal structure of the small tetraheme cytochrome (STC, *green*) and homology model of CymA (*blue*) are shown. The <100 Å distance between the tip of CymA and MtrCAB is too far to allow direct electron transfer; consequently, electrons must be transported through a series of electron shuttles, such as STC, across the periplasmic space.

The experimental scattering curves of MtrCAB show it has a maximal dimension of 174–185 Å, in agreement with MtrC (90 Å) associating with the surface of MtrAB (100 Å). Comparison of the MtrCAB and MtrAB envelopes allows the orientation of MtrAB in the membrane, with the tip of MtrAB extending ∼50 Å into the periplasm (Fig. 5). One end of MtrAB face would be exposed to the cell surface, allowing for interaction with MtrC or soluble Fe(III) citrate.

The width of the *S. oneidensis* periplasm has been measured as 235 ± 37 Å using cryo–transmission electron microscopy (28), so it is reasonable to assume that ∼200 Å represents a lower limit for the distance that electrons must travel between the inner and outer membrane of *S. oneidensis* MR-1. Homology models of CymA based on NrfH suggest that CymA could reach 50 Å across the membrane (29), and it was previously postulated that direct electron transfer between CymA and MtrA might occur (27, 30, 31). More recent studies suggested that a range of soluble cytochromes, including STC and FccA, may be required (32). Rather than electron transfer between CymA and MtrA, our proteoliposome experiments confirmed that electron transfer between MtrCAB and STC would occur at rates sufficient to sustain respiration, revealing that electron transfer between CymA and MtrCAB must occur through interactions with periplasmic cytochromes, rather than direct electron transfer between CymA and MtrCAB.

Until now there has been no experimental evidence for the position of MtrC on the surface of the lipid bilayer. Possible conformations include a parallel orientation on the surface, allowing multiple hemes to interact with both MtrAB and mineral, or a perpendicular orientation, in which case it is likely that only hemes 10 or 5 accept electrons from MtrAB (Fig. 5). The molecular envelopes of MtrCAB models generated at both concentrations show that MtrC is likely to associate with MtrAB in a conformation perpendicular to the membrane, allowing hemes 5 and 10 to function as electron ingress/egress sites.

Our structural models produced by SANS are sufficient to give the first insight into the arrangement of the component proteins of the *S. oneidensis* MtrCAB transmembrane electron channel, and how it may be arranged in the outer membrane. It is clear that MtrA inserts fully into the MtrB β-barrel, with MtrC interacting with the MtrAB surface and either heme 10 or heme 5 accepting electrons. Assuming that MtrAB is also perpendicular to the membrane then MtrC will be raised at an angle on the membrane surface, with either heme 10 or heme 5 able to donate electrons directly to an insoluble extracellular substrate.

## Experimental procedures

### Expression and purification of MtrCAB and STC from S. oneidensis MR-1

MtrCAB was isolated as a pure complex in 2% Triton X-100 as described previously (12, 23). STC was purified from *S. oneidensis* cells grown aerobically at 30 °C in Terrific Broth medium overnight. Cells were harvested at 7000 *g* and 4 °C for 20 min and re-suspended in 100 mm HEPES, pH 7.5. The cells were incubated with polymyxin B (1 mg ml^−1^) at 37 °C for 1 h before centrifugation at 15,000 *g*, 4 °C for 45 min. The supernatant was loaded onto a 15 ml HiTrap nickel affinity column (GE Healthcare) equilibrated with 20 mm HEPES, pH 7.6, and eluted via a 0–500 mm imidazole gradient over 200 ml. Fractions containing STC were pooled and dialyzed against 20 mm HEPES, pH 7.6, overnight. The dialyzed fraction was loaded onto a Q Sepharose anion exchange column, equilibrated with 20 mm HEPES, pH 7.6. After washing with 2-column volumes of buffer, STC was eluted using a linear gradient of 0–500 mm NaCl over 4-column volumes. Fractions were concentrated in an Avanti^TM^ spin concentrator before passage through a HiLoad 16/60 Superdex 75 gel filtration column equilibrated with 20 mm HEPES, pH 7.6, 100 mm NaCl. Fractions containing pure STC were spin concentrated in 20 mm HEPES buffer, pH 7.5.

### Preparation of recombinant MtrAB

Genes encoding MtrAB were cloned into a pBAD202 plasmid (Invitrogen). The genomic DNA of *S. oneidensis* MR-1 was isolated using a PureLink Genomic DNA Mini Kit (Invitrogen). The following primer pair amplified *mtrA* (1002 bp), *mtrB* (2094 bp), and their intergenic region (12 bp): forward, CACCTAAGAAGGAGATATACATCCCATGAAGAA CTGCCTAAAAATGAAAAACCTAC (overhang and start codon, respectively) and reverse, GGATTAGAGTTTGTAACTCATGCTCAGC. The forward primer contained, between the overhang and start codon, the ribosomal-binding site for the thioredoxin gene of the pBAD202 plasmid such that the native ribosomal-binding site was not used. The CACC overhang allowed for directional insertion into pBAD202/Directional TOPO. The reverse primer maintained the native stop codon of *mtrB* such that recombinant MtrAB would not include any affinity tags. PCR products were obtained with Phusion Flash High-Fidelity PCR Master Mix (Thermo Fisher Scientific) and amplification of *mtrAB* confirmed by a single 3-kb band on a 1% agarose gel. The pBAD202 Directional TOPO Expression Kit (Thermo Fisher Scientific) was used to insert amplified DNA into the pBAD202/Directional TOPO plasmid and transform chemically competent *Escherichia coli* One Shot TOP10 cells with the ligation product, pCL001. Transformed cells were streaked onto LB agar plates with kanamycin (30 μg ml^−1^) and plasmid isolated from single-colonies (Qiagen Miniprep Kit). After sequencing (Eurofins) confirmed successful plasmid construction, pCL001 was used for transformation by electroporation into an MR-1 strain lacking the mtr-gene cluster to give strain *mtr*^−^*mtrAB*^+^. Successful formation of *mtr*^−^*mtrAB*^+^ was verified through sequencing of plasmid DNA.

Recombinant MtrAB was purified from membranes of MR-1 strain *mtr*^−^*mtrAB*^+^ grown at 30 °C in M72 media (casein digest peptone 15 g liter^−1^, papaic digest of soybean meal 5 g liter^−1^, NaCl 5 g liter^−1^ at pH 7.8) supplemented with 20 mm dl-lactate, 20 mm sodium fumarate, 20 mm HEPES, pH 7.8, and 30 μg ml^−1^ kanamycin. Cultures (1.5-liter volume) were grown in 2-liter baffled flasks shaken at 200 rpm after inoculation (2%) with LB culture grown aerobically, overnight at 30 °C. The cells were induced at mid–exponential phase (*A*_600 nm_ ∼0.6) with the addition of 5 mm l-arabinose at which time shaking was stopped and the cultures left to grow microaerobically for a further 18 h. Cells were harvested by centrifugation at 6230 × *g* for 20 min at 4 °C, washed and resuspended in 20 mm HEPES, 50 mm NaCl, pH 7.8, using ∼5 ml for cells from 1 liter culture. Cell lysis was performed in the presence of lysozyme and DNase by two passes through a French press at a pressure of 16,000 psi (1 psi = 6.9 kilopascals). Cell debris and unbroken cells were removed by 30-min centrifugation at 1500 × *g*, 4 °C. Membranes were pelleted by 100-min ultracentrifugation at 200,000 × *g*, 4 °C and resuspended in 20 mm HEPES, 50 mm NaCl, pH 7.8. To preferentially solubilize inner membranes, Sarkosyl was added to the resuspended membranes to a concentration of 1% (m/v) and the suspension stirred gently at 4 °C for 45 min. The pellet recovered from a second round of ultracentrifugation was then solubilized by stirring overnight in 20 mm HEPES, 50 mm NaCl, pH 7.8 containing 5% (v/v) Triton X-100. Insoluble material was removed by a final round of ultracentrifugation and recombinant MtrAB purified from the supernatant by anion exchange chromatography at 4 °C, as below, monitored by electronic absorbance spectroscopy and SDS-PAGE with proteins visualized by heme and Coomassie Blue stain.

Solubilized membrane proteins were loaded (2 ml min^−1^) onto a Q Sepharose column (300 ml) pre-equilibrated with Buffer A (20 mm HEPES, 50 mm NaCl, pH 7.8, 2% (v/v) Triton X-100). After washing the column with Buffer A until a stable baseline was achieved (monitored by the absorbance at 280 nm), the bound proteins were eluted with a gradient of 0–50% Buffer B (Buffer A plus 1 m NaCl) over 800 ml (50–525 mm NaCl) while collecting 10 ml fractions. The purest MtrAB-containing fractions were pooled, diluted into 20 mm Tris-HCl, 50 mm NaCl, pH 8.5, containing 2% (v/v) Triton X-100 (Buffer C) and loaded on to a DEAE column (300 ml) pre-equilibrated with Buffer C. Bound protein was washed at 2 ml min^−1^ with Buffer C and eluted with a gradient from 0–50% Buffer D (Buffer C plus 1 m NaCl) over 800 ml (50–525 mm NaCl) while collecting 10 ml fractions. Fractions containing the purest MtrAB were pooled, diluted 3-fold with 5 mm lauryldimethylamine *N*-oxide, 20 mm HEPES, 50 mm NaCl, pH 7.8 (Buffer E) and loaded onto a HiTrap Q Sepharose column pre-equilibrated with Buffer E. The column was washed with 2- column volumes of Buffer E and eluted with Buffer F (Buffer E plus 300 mm NaCl). The resulting sample was concentrated and exchanged into Buffer E with a Microcon-30kDa Centrifugal Filter (Millipore Sigma) and stored as frozen aliquots at −80 °C.

### Preparation of liposomes and proteoliposomes

Liposomes and MtrCAB proteoliposomes containing internalized methyl viologen were prepared according to previously published methods (12, 25). Liposomes and MtrCAB proteoliposomes containing STC were prepared through the following procedure. A 10 mg/ml phosphatidylcholine (Sigma-Aldrich) suspension prepared in 20 mm HEPES, pH 7.5, 100 mm NaCl, and 2.6 mg/ml STC was extruded though a 0.1 μm polycarbonate membrane and frozen at −80**°**C. After thawing, the suspension was sonicated twice for 40–45 s. Valinomycin and MtrCAB were added to give final concentrations of 100 nm and 10 nm, respectively, and the suspension was subjected to two freeze thaw cycles. 0.25 g of BioBeads (Bio-Rad) were added to the sample and incubated at 4**°**C for 1 h to remove detergent. The supernatant was ultracentrifuged at 280,000 *g* for 45 min to isolate the proteoliposomes. The supernatant containing the excess cytochrome was removed and the proteoliposome/liposome pellet gently resuspended in 1 ml of fresh 20 mm HEPES pH 7.5, 100 mm NaCl, 2 mm CaCl_2_, 10 mm KCl buffer. The absorbance of the supernatant was measured and centrifugation at 280,000 *g* for 45 min followed by supernatant removal, followed by pellet resuspension was repeated until the Soret peak of excess soluble cytochrome was no longer visible in the supernatant.

### Spectrophotometric analysis of proteoliposomes

100 to 300 μl of proteoliposome stock suspension was added to 3 ml of 20 mm HEPES, pH 7.5, 100 mm NaCl, 2 mm CaCl_2_, 10 mm KCl buffer. The diluted proteoliposome/liposome suspension was transferred to an anaerobic quartz cuvette and flushed with argon for 1–2 h with gentle stirring. Reduction of liposomes and proteoliposomes was performed by addition of 15 μl of a 17 mm solution of reduced sodium dithionite. Oxidation of proteoliposomes was performed by addition of 175–300 μm Fe(III) citrate, freshly prepared from ferric ammonium citrate (Sigma) or 1 mm potassium ferricyanide to proteoliposomes that had been carefully reduced by titrating 2 μl aliquots of a 17 mm solution of reduced sodium dithionite. The absorbance of the proteoliposomes was monitored using a Hitachi U-4100 UV-visible spectrophotometer. Wavelength scans were taken before and after time scans tracking the absorbance at 552 nm for STC and 603 nm for methyl viologen proteoliposomes.

### Analytical ultracentrifugation

Sedimentation velocity experiments were performed at 35,000 rpm, using a Beckman Optima analytical ultracentrifuge with an An-50 Ti rotor and at 20 °C. 0.5 μm MtrCAB was diluted into 20 mm HEPES, pH 7.8,100 mm NaCl, 2.8 mm Fos–choline-12, 51% D_2_O. 51% D_2_O was used to match the buffer density to the density of the Fos–choline micelles. Data were recorded using the absorbance (at 410 nm with 10 μm resolution and recording scans every 20 s) optical detection system. The density and viscosity of the buffer was measured experimentally using a DMA 5000 m densitometer equipped with a Lovis 200ME viscometer module. The partial specific volume of the protein complex was calculated using SEDNTERP from the amino acid sequence. Data were processed using SEDFIT, fitting to the c(s) model, and in SEDPHAT fitting to the hybrid global c(s) global discrete species model.

### SANS data collection and analysis of MtrCAB and MtrAB

SANS is used to obtain structural information on membrane proteins, as proteins and detergent molecules scatter radiated neutrons differently, resulting in different scattering length densities. This enables “contrast matching” where the scattering length of the solvent is adjusted to match the scattering length of the detergent, which is achieved by altering the ratio of D_2_O/H_2_O in the solvent. At the contrast match point, the scattering from detergent micelles is identical to the solvent scattering so after solvent subtraction the scattering of the protein is obtained. Structural information about the protein is therefore derived independently of the contributions from the supporting detergent micelle. The literature value for the contrast match point of Fos–choline-12 was previously published as 11% (33, 34). A similar value of 13% had previously been obtained on beamline D22 (ILL), and this value was subsequently used as the contrast match point for samples of MtrCAB and MtrAB solubilized in Fos–choline-12.

MtrCAB and MtrAB were purified as described in supporting data and concentrated to ∼15 mg/ml using a 100,000 molecular weight cut-off spin concentrator (Millipore) and dialyzed overnight in a sealed DURAN bottle containing 20 mm HEPES, pH 7.8, plus 100 mm NaCl plus 13% D_2_O plus 2.8 mm Fos–choline-12 using a 50,000 molecular weight cut-off Dispo-Biodialyzer (Sigma-Aldrich). Protein concentrations were determined by UV-visible spectroscopy utilizing the heme Soret band absorbance at 410 nm (single heme extinction coefficient: 110,000 m^−1^ cm^−1^) and diluted to the required concentration (MtrCAB: 8.7 mg/ml and 3.5 mg/ml, MtrAB: 9.7 mg/ml and 3.5 mg/ml) made using the dialysis buffer. 200 μl samples were centrifuged at 13,000 × *g* for 10 min at 4 °C before being placed in 0.1 cm path-length Suprasil quartz cuvette (Hellma) and sealed with parafilm.

Initial small-angle neutron scattering characterization was carried out on SANS2D (ISIS, Oxfordshire, UK). Monodispersity of the samples was confirmed via Guinier analysis over a range of concentrations and used to optimize both buffer and detergent conditions.

Further small-angle neutron scattering data were collected on D22 diffractometer of ILL (Grenoble, France) using two configurations of collimation 8 m and 2.8 m, and detector distance 8 m and 1.4 m, respectively. The collimation cross-section was 40 mm × 55 mm and the sample aperture was 7 mm × 10 mm. The exposure times ranged from 60 s to 1 h depending on sample concentration, contrast, and instrument configuration. Data reduction using GRASP software included blocked beam and empty cell background subtraction, scaling for sample thickness and transmission, calibration to absolute intensity using incident neutron flux at sample position, and finally, azimuthal averaging.

Merging of curves recorded at different instrumental configurations and buffer subtraction were done using NCNR macros for Igor Pro (WaveMetrics). Data analysis was performed using the ATSAS program suite (35). Within the ATSAS suite, the program PRIMUS (36) was used to calculate the radius of gyration (*R_g_*) and Kratky plots to evaluate disorder within the sample. The program GNOM (20) was used to plot the *P*(*r*) function from the Fourier inversion of the scattering intensity, *I*(*Q*). The *P*(*r*) function was used to calculate the *R_g_* and maximum particle size (*D*_max_), and also for the reconstruction of *ab initio* envelopes by the application of 20 cycles in DAMMIN (37). The resulting bead models were sequentially analyzed using DAMSEL, DAMSUP, and DAMAVER to compare and identify the most probable model, align all models to the most probable model, average these aligned models and compute a probability map with the averaged model then filtered using DAMFILT (11). The averaged, filtered model was refined against the experimental data to final models to the data.

## Author contributions

M. J. E., A. M., and T. A. C. data curation; M. J. E., G. F. W., C. W. L., M. L., A. M., G. H., D. J. S., J. N. B., and T. A. C. formal analysis; M. J. E., G. F. W., M. L., D. J. S., and T. A. C. validation; M. J. E., G. F. W., C. W. L., M. L., A. M., G. H., D. J. S., J. N. B., and T. A. C. investigation; M. J. E., G. F. W., and T. A. C. visualization; M. J. E., G. F. W., C. W. L., M. L., G. H., D. J. S., J. N. B., and T. A. C. methodology; M. J. E., C. W. L., D. R., J. N. B., and T. A. C. writing-original draft; M. J. E., A. M., D. J. S., D. R., J. N. B., and T. A. C. writing-review and editing; G. F. W., A. M., D. J. S., J. N. B., and T. A. C. supervision; A. M. resources; A. M. and D. J. S. software; D. J. S., D. R., and T. A. C. funding acquisition; D. J. S., D. R., J. N. B., and T. A. C. project administration; D. R. and J. N. B. conceptualization.

## Supplementary Material

Supporting Information

## Footnotes

3 The abbreviations used are:

STC: small tetraheme cytochrome
SANS: small angle neutron scattering
*R_g_*: radius of gyration
*D*_max_: maximum distance
Buffer A: 20 mm HEPES, 50 mm NaCl pH 7.8, 2% (v/v) Triton X-100
Buffer B: Buffer A plus 1 m NaCl
Buffer C: 20 mm Tris-HCl, 50 mm NaCl, pH 8.5, containing 2% (v/v) Triton X-100
Buffer D: Buffer C plus 1 m NaCl
Buffer E: 5 mm lauryldimethylamine *N*-oxide, 20 mm HEPES, 50 mm NaCl, pH 7.8
Buffer F: Buffer E plus 300 mm NaCl.
