# Supplementary material for: Structural modeling of an outer membrane electron conduit from a metal-reducing bacterium suggests electron transfer via periplasmic redox partners
Source: J Biol Chem. 2018 Apr 10;293(21):8103–12. doi: 10.1074/jbc.RA118.001850 (PMC5971433; doi:10.1074/jbc.RA118.001850)
Supplement: Supporting Information [file supp_293_21_8103__index.html]

Structural model of a porin-cytochrome electron conduit from the outer membrane of a metal reducing bacterium suggests electron transfer via periplasmic redox partners. — Solution structure of MtrCAB. — Structural modeling of an outer membrane electron conduit from a metal-reducing bacterium suggests electron transfer via periplasmic redox partners — Solution structure of MtrCAB — Supporting Information 

# Structural modeling of an outer membrane electron conduit from a metal-reducing bacterium suggests electron transfer via periplasmic redox partners

## Supporting Information

- Supporting information - Supplemental figures and tables.
