# Supplementary material for: Structural modeling of an outer membrane electron conduit from a metal-reducing bacterium suggests electron transfer via periplasmic redox partners
Source: J Biol Chem. 2018 Apr 10;293(21):8103–12. doi: 10.1074/jbc.RA118.001850 (PMC5971433; doi:10.1074/jbc.RA118.001850)
Supplement: Supporting Information [file supp_RA118.001850_135217_1_supp_98854_p5pp8x.docx]

Supplemental Table S1: Small angle neutron scattering analysis of *S. oneidensis* MR-1 MtrAB and MtrCAB in 20 mM HEPES pH 7.8, 100 mM NaCl, 2.8 mM Fos-Choline-12, 13 % D_2_O. Samples were measured and analysed as described in methods using PRIMUS and GNOM.

| Sample |  | MtrAB | | | MtrCAB | |
| --- | --- | --- | --- | --- | --- | --- |
| Dataset |  | 3.5 | 8.7 | Merged | 3.5 | 9.7 |
| **Data-collection parameters** | |  |  |  |  |  |
| Wavelength, (Å) | | 6 | 6 | 6 | 6 | 6 |
| q range, (Å^−1^) | | 0.007-0.15 | 0.008-0.16 | 0.007-0.16 | 0.007-0.15 | 0.007-0.15 |
| Exposure time (s) | | 3600 | 3600 |  | 3600 | 3600 |
| Concentration range (mg·ml^−1^) | | 3.5 | 8.7 | 3.5-8.7 | 3.5 | 9.7 |
| Temperature (K) | | 10 | 10 |  | 10 | 10 |
| **Structural parameters** | |  |  |  |  |  |
| Guinier I(0) | | 0.09 | 0.22 | 0.22 | 0.19 | 0.57 |
| Guinier Rg, (Å) | | 29.2 ± 2.6 | 30.4±1.2 | 29.4 ± 2 | 53.7 ± 5.4 | 53.1±2.5 |
| I(0) [from P(r)] | | 0.09 | 0.22 | 0.22 | 0.18 | 0.56 |
| Rg, Å [from P(r)] | | 30.2 ± 0.7 | 29.9 ± 0.5 | 30.3 ± 0.4 | 53.7 ± 0.2 | 52.7 ± 0.6 |
| Dmax (Å) |  | 105 | 109 | 107 | 171 | 185 |
| M_w_ from I(0) (Da) | | 104619 | 102882 | 134819 | 183538 | 206033 |
| M_w_ from sequence (Da) | | 114045 | 114045 | 114045 | 189280 | 189280 |
|  |  |  |  |  |  |  |

Supplemental Table S2: *Ab Initio* modelling of MtrAB and MtrCAB data. GNOM was used to fit experimental data to distance distribution curves with different of D_max_ values. The Estimate is a total estimate of the quality of the p(r) distribution fit to experimental data. Estimates that were greater than 5% lower than the highest Estimate value were discounted. DAMSEL was used to align 20 models generated using experimental data while DAMMIN refined a core structure to the data giving an overall chi2 estimate.

| **Program** | **Gnom** |  |  | **Damsel** | | **Dammin** | | |
| --- | --- | --- | --- | --- | --- | --- | --- | --- |
| Data | Dmax | Rg | Estimate | NSD | Models | chi^2^ | Dmax | Rg |
| **MtrAB** |  |  |  |  |  |  |  |  |

| 3.5 - 8.7 | 98.3 | 29.7 ± 0.6 | 0.5724 |  |  |  |  |  |
| --- | --- | --- | --- | --- | --- | --- | --- | --- |
| 3.5 - 8.7 | 101.3 | 29.5 ± 0.7 | 0.6438 | 0.539 ± 0.032 | 17 | 0.3785 | 101.4 | 29.78 |
| **3.5 - 8.7** | **104.3** | **29.7 ± 0.5** | **0.6871** | **0.530 ± 0.024** | **18** | **0.5484** | **102.1** | **30.18** |
| 3.5 - 8.7 | 107.3 | 30.0 ± 0.4 | 0.6850 | 0.458 ± 0.01 | 19 | 0.3601 | 101.9 | 29.56 |

| 3.5 - 8.7 | 110.3 | 29.5 ± 1.1 | 0.6296 |  |  |  |  |  |
| --- | --- | --- | --- | --- | --- | --- | --- | --- |
| **MtrCAB** |  |  |  |  |  |  |  |  |
| 3.5 | 153 | 49.4±0.6 | 0.5846 |  |  |  |  |  |

| 3.5 | 160 | 52.1±0.2 | 0.4959 |  |  |  |  |  |
| --- | --- | --- | --- | --- | --- | --- | --- | --- |
| 3.5 | 166.5 | 52.9 ± 1.9 | 0.4939 |  |  |  |  |  |
| 3.5 | **170.5** | **52 ± 1.2** | **0.6713** | **0.681 ± .045** | **19** | **0.693** | **181** | **52.9** |
| 3.5 | 174.5 | 52.6 ± 1.5 | 0.6577 | 0.712 ± 0.075 | 19 | 0.6779 | 186.6 | 52.99 |
| 3.5 | 178.5 | 53.0 ± 1.5 | 0.6449 | 0.667 ± 0.043 | 19 | 0.6791 | 187 | 53.1 |
| 3.5 | 182.5 | 53.4 ± 1.6 | 0.6392 | 0.704 ± 0.063 | 19 | 0.6742 | 187.3 | 53.6 |
| 3.5 | 186.5 | 53.9±1.6 | 0.6263 |  |  |  |  |  |

| 9.7 | 155 | 50±0.33 | 0.568 |  |  |  |  |  |
| --- | --- | --- | --- | --- | --- | --- | --- | --- |

| 9.7 | 160 | 51.2±0.7 | 0.5852 |  |  |  |  |  |
| --- | --- | --- | --- | --- | --- | --- | --- | --- |
| 9.7 | 165 | 51.6±0.6 | 0.7070 | 0.821±0.1 | 19 | 0.637 | 186.1 | 52.01 |
| 9.7 | **170** | **51.9 ± 0.8** | **0.6894** | **0.724±0.07** | **18** | **0.638** | **185.1** | **52.02** |
| 9.7 | 175 | 52.1±0.8 | 0.6796 | 0.657±0.04 | 18 | 0.624 | 186.4 | 52.33 |
| 9.7 | 180 | 52.4±08 | 0.6613 | 0.668±0.08 | 18 | 0.6165 | 186.8 | 52.55 |

| 9.7 | 185 | 52.8±0.9 | 0.5071 |  |  |  |  |  |
| --- | --- | --- | --- | --- | --- | --- | --- | --- |

Supplemental Figure S1: Putative structure of the MtrCAB complex. **(A)** Cartoon diagram showing the possible arrangement of MtrC, MtrB and MtrA in the outer membrane. **(B)** X-ray crystal structure of MtrC showing the positions of hemes 5 and 10. Image generated using MtrC structure PDB ID: 4LM8


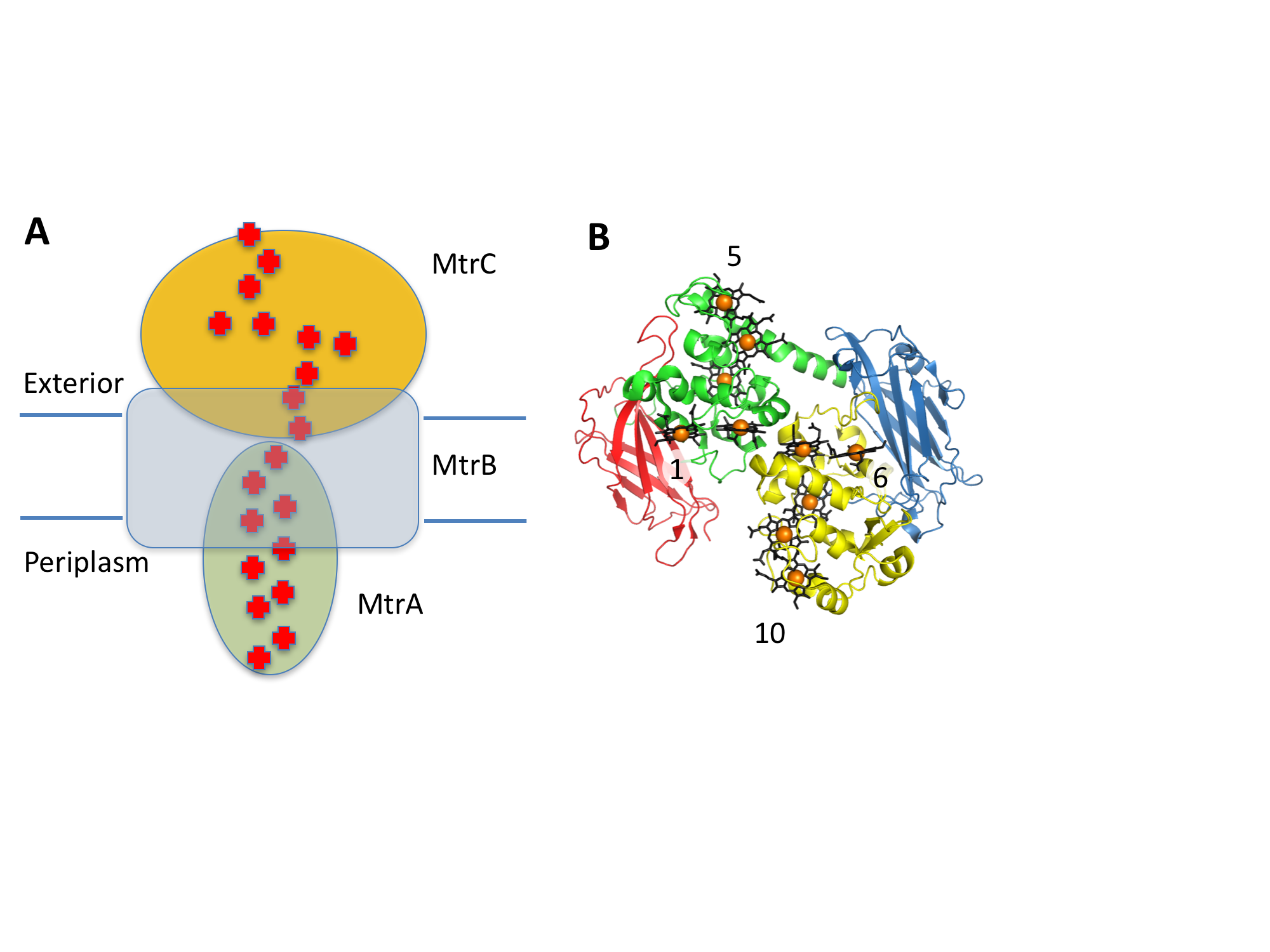


Supplemental figure S2: 12% SDS-polyacrylamide gels stained with Coomassie of (A) MtrCAB and (B) MtrAB samples purified in fos-choline 12.

Supplemental Figure S3: Molecular envelope of MtrAB. **Top**: Final fit of DAMMIN model to MtrAB merged scattering data. **Bottom**: Final Molecular envelope generated by DAMMIN.

Supplemental figure S4: Sedimentation velocity of MtrCAB in 20 mM Hepes pH 7.8 ,100 mM NaCl, 2.8 mM Fos-Choline-12, 51 % D_2_O. Data were processed using SEDFIT, fitting to the c(s) model, and in SEDPHAT fitting to the hybrid global c(s) global discrete species model. The experimental fit to the raw data is shown in (A), while the c(s) distribution between 0 -18 S is shown in (B), revealing a major sedimenting peak corresponding to MtrCAB.


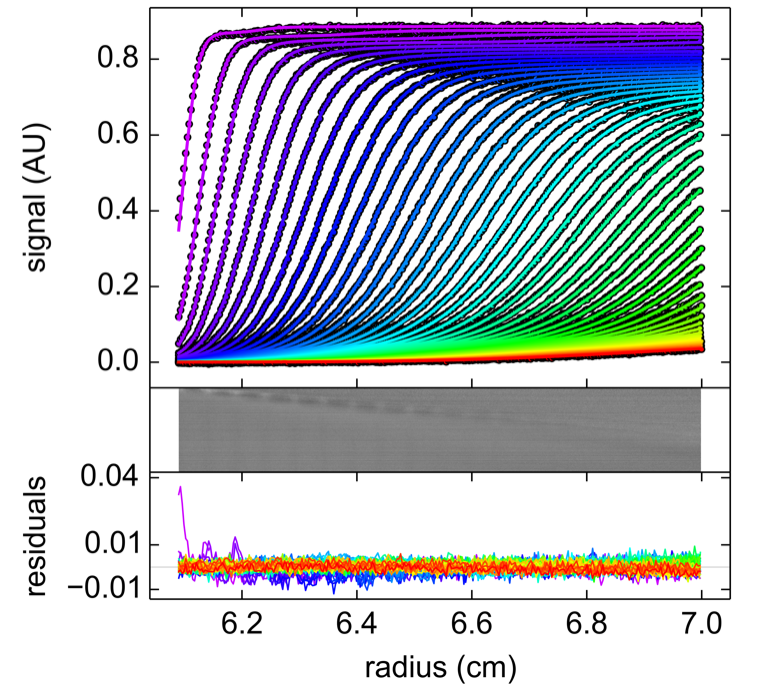


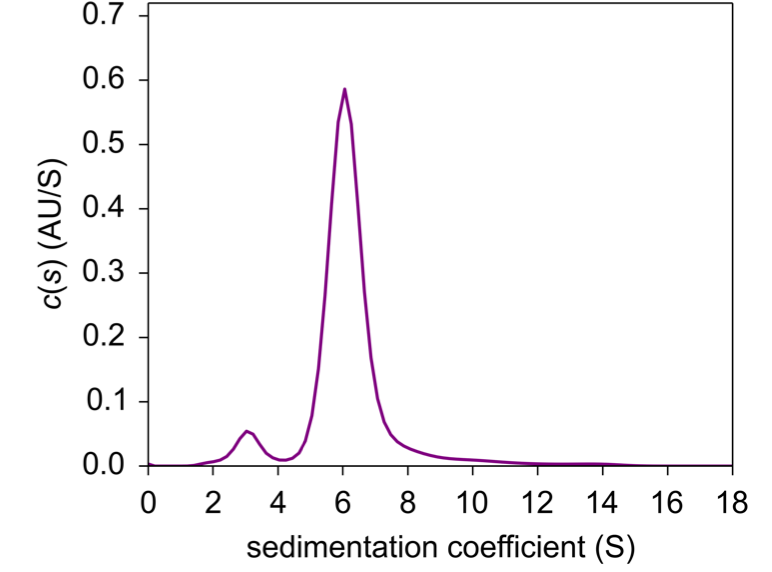


Supplemental figure S5: Cartoon showing oxidation and reduction of proteoliposomes. **(A)** MtrCAB proteoliposomes containing internal STC and membrane embedded MtrCAB. Addition of sodium dithionite reduces the internalized STC, which can then be oxidized by addition of potassium ferricyanide. **(B)** Addition of sodium dithionite to proteoliposomes containing STC but no MtrCAB did not reduce the internalized STC as there was no mechanism for electron transport across the lipid bilayer.
